# Exploring Unlabeled Data in Multiple Aspects for Semi-Supervised MRI Segmentation

**DOI:** 10.34133/hds.0166

**Published:** 2024-08-05

**Authors:** Qingyuan He, Kun Yan, Qipeng Luo, Duan Yi, Ping Wang, Hongbin Han, Defeng Liu

**Affiliations:** ^1^ Radiology Department, Peking University Third Hospital, Beijing, China.; ^2^ Peking University Third Hospital, Beijing Key Laboratory of Magnetic Resonance Imaging Devices and Technology, Beijing, China.; ^3^ School of Computer Science, Peking University, Beijing, China.; ^4^ Department of Pain Medicine, Peking University Third Hospital, Beijing, China.; ^5^ School of Software and Microelectronics, Peking University, Beijing, China.; ^6^ National Engineering Research Center for Software Engineering, Peking University, Beijing, China.; ^7^ Key Laboratory of High Confidence Software Technologies (Peking University), Ministry of Education, Beijing, China.; ^8^ Department of Obstetrics and Gynecology, Peking University Third Hospital, Beijing, China.; ^9^ Center for Reproductive Medicine, Peking University Third Hospital, Beijing, China.

## Abstract

**Background:** MRI segmentation offers crucial insights for automatic analysis. Although deep learning-based segmentation methods have attained cutting-edge performance, their efficacy heavily relies on vast sets of meticulously annotated data. **Methods:** In this study, we propose a novel semi-supervised MRI segmentation model that is able to explore unlabeled data in multiple aspects based on various semi-supervised learning technologies. **Results:** We compared the performance of our proposed method with other deep learning-based methods on 2 public datasets, and the results demonstrated that we have achieved Dice scores of 90.3% and 89.4% on the LA and ACDC datasets, respectively. **Conclusions:** We explored the synergy of various semi-supervised learning technologies for MRI segmentation, and our investigation will inspire research that focuses on designing MRI segmentation models.

## Introduction

MRI (magnetic resonance imaging) segmentation refers to the process of partitioning or dividing MR images into different regions or structures based on their characteristics, such as tissue types, anatomy, or pathological features. This precise segmentation offers crucial insights to clinicians for accurate diagnosis, disease tracking, and treatment planning. Recent advancements in neural networks have led deep learning-based approaches to achieve state-of-the-art performance in various MRI segmentation tasks [1–4].

However, the efficacy of deep learning approaches hinges on large-scale dense annotations, which are extremely expensive and labor-consuming to obtain. This presents an important barrier to implement deep learning-based MRI segmentation models in real-world medical settings. Such a challenge has spurred research into semi-supervised techniques, which aim to train models using a small amount of labeled data combined with abundant unlabeled data, while still achieving satisfactory performance [5–16].

Within the realm of semi-supervised learning for MRI segmentation, advancements have given rise to 2 primary streams, namely, pseudo-labeling [5,6,13] and consistency regularization [7,8,10]. Pseudo-labeling methods optimize models by extrapolating from a limited set of labeled data to generate pseudo-labels for the unlabeled data. These labels are then amalgamated with the labeled dataset for model refinement. On the other hand, consistency regularization techniques enforce consistency between predictions of different perturbations, such as data augmentations [7] and model perturbing [8]. While pseudo-labeling harnesses information from unlabeled data, consistency regularization bolsters the stability and generalization of deep learning models. However, the synergy between these technologies—integrating pseudo-labeling and consistency regularization to maximize the utilization of unlabeled data—remains an underexplored territory in MRI segmentation, not to mention some other semi-supervised learning technologies, such as those based on generative networks [17,18]. Efforts to fully leverage the complementary strengths of various semi-supervised learning technologies for optimized utilization of unlabeled data in MRI segmentation require further exploration and investigation.

Furthermore, while there exists a range of semi-supervised MRI segmentation models, they predominantly adopt an encoder–decoder architecture. Therefore, in this study, we introduce an innovative and adaptable training approach for semi-supervised MRI segmentation. This approach offers dual benefits: (a) First, it merges the strengths of various semi-supervised learning techniques, fostering their synergistic influence on model refinement. (b) Second, it is designed to seamlessly integrate with encoder–decoder-based semi-supervised MRI segmentation models, ultimately enhancing their performance.

## Methods

### MRI datasets

In this study, we validated our method using 2 public MRI segmentation datasets [19,20]. The requirement for written informed consent from enrolled patients was waived due to the retrospective design of the study.

The Left Atrial (LA) dataset: The LA dataset [19] was obtained from the 2018 Left Atrium Segmentation Challenge (https://www.cardiacatlas.org/atriaseg2018-challenge/atria-seg-data/) and it includes 100 three-dimensional (3D) gadolinium-enhanced MRI scans collected by 2 clinical MR scanners (Avanto 1.5T and Verio 3.0T, Siemens, Germany) in patients with atrial fibrillation (AF) ranging in time from 3 to 27 months after clinical ablation at the University of Utah. As provided by the competition organizing committee, 3D MRI scans have a spatial resolution of 0.625 × 0.625 × 0.625 mm^3^ and a spatial size of 576 × 576 × 88 pixels or 640 × 640 × 88 pixels. The LA cavity is defined as the pixels within the surface of the LA endocardium, including the mitral valve, the LA auricles, and the sleeve extent of the pulmonary veins (PVs). Three trained observers performed manual segmentation of each late gadolinium enhancement MRI (LGE-MRI) scan using Corview image processing software [Merrk Inc., Salt Lake City, UT (McGann et al., 2014)]. The grayscale LGE-MRI image volumes and associated binary LA segmentations were stored in the nearly raw raster data (nrrd) format. Following previous studies [8–10], the dataset was split into a training set and a testing set at a ratio of 8:2. In the training dataset, the labeled proportion *p* was set to 10% or 20%, and the corresponding number of patients randomly selected as labeled patients with images and labels available (16 patients for *p* = 20%, 8 patients for *p* = 10%, all slices within one patient were simultaneously set to labeled or unlabeled), while others as unlabeled patients with only images available for training. Given that the existing research did not separate the validation set, we took the initiative to divide the initial training set into a fresh training set and a dedicated validation set. Initially, we trained the model using various configurations (e.g., *p* = 10% or 20%) based on this new training data. Subsequently, we assessed the model’s performance using the validation set. Upon determining the optimal hyperparameters from this validation process, we retrained the model using these parameters on the original training data. Finally, we rigorously tested our model on the designated testing set to ensure a fair and comprehensive comparison with other existing models.

The ACDC dataset: The ACDC dataset [20] was sourced from the 2017 Automated Cardiac Diagnosis Challenge (ACDC) (https://www.creatis.insa-lyon.fr/Challenge/acdc/databases.html). This dataset contains MR images of 100 subjects, including 20 healthy volunteers and 80 patients divided into 5 subgroups on average: previous myocardial infarction, dilated cardiomyopathy, hypertrophic cardiomyopathy, and abnormal right ventricle. Each group was distinctly defined based on physiological parameters, including left or right diastolic volume, ejection fraction, local contraction of the left ventricle (LV), LV mass, and maximum thickness of the myocardium. Acquisitions were performed using 2 MRI scanners with different magnetic field strengths (Area 1.5T, Siemens, Germany and Trio Tim 3.0T, Siemens, Germany) over a period of 6 years in the Hospital of Dijon (France). Cinematic MR images were acquired retrospectively or prospectively gated under breath-hold using conventional steady-state free procession (SSFP) sequences. After acquisition of long-axis slices, a series of short-axis slices were acquired covering the LV from base to apex, with slice thicknesses ranging from 5 to 10 mm (usually 5 mm). Spatial resolution varied from 1.34 to 1.68 mm^2^/pixel. Depending on the patient, 28 to 40 volumes were acquired to completely cover (retrospectively gated) or partially cover (prospectively gated) one cardiac cycle. The complete dataset was acquired during a clinical routine, which resulted in natural variations in image quality, field-of-view variations, and overall or nearly overall coverage of the LV. The dataset was divided into the training set, validation set, and testing set at a ratio of 7:1:2, following previous studies [6]. In the training dataset, the labeled proportion *p* was set to 5% or 10%, and the corresponding number of patients was selected as labeled patients with images and labels available (7 patients for *p* = 10%, 3 patients for *p* = 5%, all slices within one patient were simultaneously set to labeled or unlabeled), while others as unlabeled patients with only images available for training.

### Method overview

**Fig. 1.**
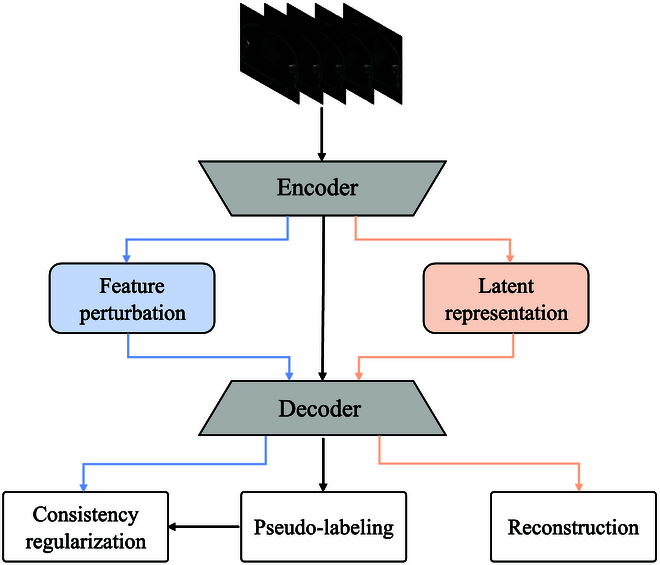
Overview of our method. It consists of 3 branches, i.e., consistency regularization, pseudo-labeling, and reconstruction.

The overall architecture of our method is shown in Fig. 1. It employs 3 strategies to capitalize on unlabeled data within existing encoder–decoder-based MRI segmentation models. Initially, pseudo-labels are created by leveraging predictions from unlabeled data, facilitating model refinement using both labeled and unlabeled datasets. Second, feature perturbation is applied to the input features directed to the decoder, ensuring output consistency with the corresponding pseudo-label. Last, to cultivate richer feature representations, an auxiliary reconstruction task is introduced.

Here, we elaborate on each step to delineate their specific functionalities in the following sections.

### Pseudo-labeling branch

We denote the input MRI volumes as *X* , which comprises labeled data *X_L_* and unlabeled data *X_U_*, i.e., *X* = {*X_L_*, *X_U_*}. Correspondingly, The ground-truth labels for *X* are denoted as *Y* = {*Y_L_*, *Y_U_*}. *Y* is voxel-wise, and each element in *Y* represents a manually annotated segmentation map corresponding in shape to its input volume. Notably, *Y_U_* denotes the manually annotated segmentation for *X_U_*, which is not available during training.

In the pseudo-labeling branch (as shown by the black arrow in Fig. 1), we apply the standard cross-entropy loss on both labeled and unlabeled data, and the Dice loss [21] is also used on the labeled data. Concretely, assuming that there are *N*_1_ and *N*_2_ MRI slices in *X_L_* and *X_U_*, respectively, the total loss from pseudo-labeling, denoted as $L_{p}$, is derived by summing the supervised loss $L_{s}$ on labeled slices and the unsupervised loss $L_{u}$ on unlabeled slices. $L_{s}$ integrates the standard cross-entropy loss and Dice loss, expressed as follows:

$$\begin{array}{c} L_{S}=\frac{1}{\mathrm{HW}N_{1}}\sum_{n=1}^{N_{1}}‍\sum_{i=1}^{\mathrm{HW}}‍H\left(Y_{L_{n}}^{i},P_{n}^{i}\right)+\\ \frac{1}{N_{1}}\sum_{n=1}^{N_{1}}‍G\left(Y_{L_{n}},P_{n}\right) \end{array}$$ (1)

where $H\left(\cdot ,\cdot \right)$ represents the standard cross-entropy function, while $G\left(\cdot ,\cdot \right)$ symbolizes the Dice loss. *H* and *W* denote the height and the width of the input, respectively. Additionally, $P_{n}^{i}$ indicates the predicted value at the pixel of *i* in the *n*th labeled slice, and $Y_{L_{n}}^{\left(i,j\right)}$ is the corresponding ground truth.

$L_{u}$ denotes the unsupervised loss and is a variant of the cross-entropy loss, which can be defined as follows:

$$L_{U}=\frac{1}{\mathrm{HW}N_{2}}\sum_{n=1}^{N_{2}}‍\sum_{i=1}^{\mathrm{HW}}‍1_{\left[\max\left(P_{n}^{i}\right)\geq \tau_{1}\right]}H\left({\hat{Y_{U}}}_{n}^{i},P_{n}^{i}\right)$$ (2)

where 1_[·]_ represents the indicator function that excludes predictions with maximal confidences below the pre-set threshold *τ*_1_. The term $P_{n}^{i}$ indicates the prediction at the pixel of *i* in the *n*th unlabeled slice, while ${\hat{Y_{U}}}_{n}^{i}=\text{argmax}\left(P_{n}^{i}\right)$ corresponds to the derived one-hot pseudo-label. The threshold *τ*_1_ is set to 0.9 by default to ensure the reliability of the pseudo-labels generated.

### Consistency regularization branch

**Fig. 2.**
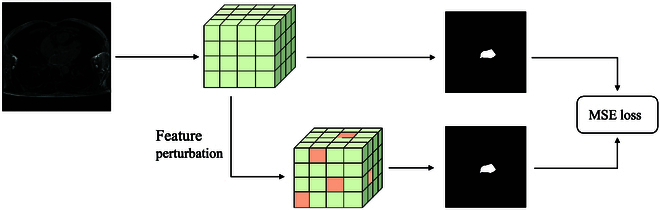
Illustration of our consistency regularization branch.

To impose consistency constraints, illustrated by the blue arrow in Fig. 1, we introduce perturbations to the features supplied to the decoder. Subsequently, we align predictions made on these perturbed features with the predictions derived from the original, undisturbed features. Specifically, as depicted in Fig. 2 and given *N* slices, the formulation of consistency regularization is expressed as:

$$L_{C}=\frac{1}{N}\sum_{n=1}^{N}‍{\left({\hat{Y}}_{n},{\tilde{Y}}_{n}\right)}^{2}$$ (3)

$${\hat{Y}}_{n}=\text{argmax}\left(h\left(I_{n}\right)\right)$$ (4)

$${\tilde{Y}}_{n}=\text{argmax}\left(h\left(g\left(I_{n}\right)\right)\right)$$ (5)

where *h*(·) represents the convolutional layer preceding the prediction within the decoder, *I_n_* signifies the feature map fed into *h*(·), and *g*(·) denotes the perturbation function that introduces noise, drawn from a standard normal distribution, to each element in *I_n_* with a possibility of 5%.

### Reconstruction branch

**Fig. 3.**
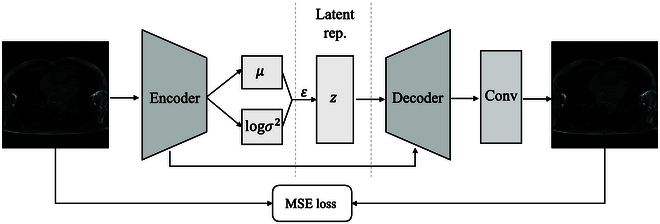
The overall pipeline of our reconstruction branch. Latent rep., latent representation; Conv, convolutional layer.

In recent years, deep generative methods have emerged as powerful tools for exploring the underlying distribution of training samples. Taking inspiration from the conditional variational auto-encoder (cVAE) [17] framework, we introduce an auxiliary reconstruction branch aimed at learning meaningful representations. As illustrated in Fig. 1, the encoder initially maps input data to a latent space. Subsequently, the decoder uses these latent space representations to reconstruct the input data. Specifically, as depicted in Fig. 3, the latent variable *z* is derived from the approximate posterior distribution *q*(*z*| *X*), which takes *μ* and *σ* as inputs, where *μ* and *σ* denote the mean and SD, respectively. Besides, as auxiliary noise variable *ϵ* is sampled from a standard normal distribution, the final computation of *z* is computed as:

z=μ+σ⊙ϵ (6)

Utilizing the output of the encoder, denoted as E*(X)*, we derive *μ* and log(*σ*^2^) through 2 distinct fully connected neural network layers, independently processing the input E*(X)*. Subsequently, the decoder takes *z* and E*(X)* as inputs, wherein E*(X)* undergoes reshaping and concatenation with *z*. Ultimately, the reconstruction is generated by passing the output of the decoder through a convolutional layer. Additionally, we also adopt the same mean squared error (MSE) loss in Eq. 3 to constrain predictions.

### Teacher–student architecture

**Fig. 4.**
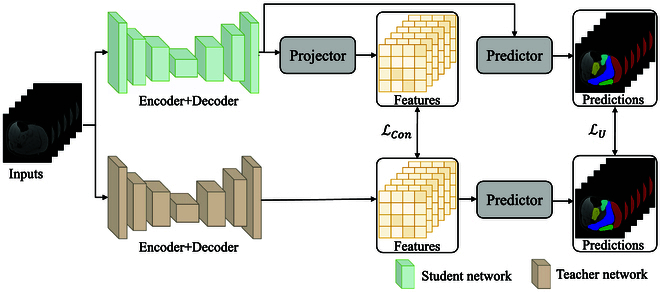
Overview of the teacher–student architecture for our approach.

The teacher–student network, a highly regarded model architecture, is widely used for semi-supervised segmentation tasks. [7,22–24]. Building upon the framework of CTSSeg [7], we tailor a teacher–student architecture for our approach, as depicted in Fig. 4. Both the teacher and student networks share the same underlying structure, with the exception that the student network incorporates a Projector—a convolutional layer designed to manipulate features. In contrast to the student network, the teacher network updates its parameters (*ϕ*) not through gradient back-propagation but by using an exponential moving average of the student’s parameters *θ* [25]. Specifically, with a decay rate *η* ranging from 0 to 1, the parameter updates for the teacher network are defined as follows:

$$\phi_{i}=\eta \phi_{i-1}+\left(1-\eta \right)\theta_{i}$$ (7)

where *i* denotes the *i*th iteration. By default, we set *η* = 0.95 following [7].

It should be noted that within this teacher–student architecture, the learning process of the student network is guided by pseudo-labels generated by the teacher network.

### Implementation details

Input processing: For the LA dataset, the raw images were converted into a 3-channel PNG format, with spatial resolutions of either 576 × 576 or 640 × 640, and a *Z* direction depth (slice count) of 88. For the ACDC dataset, the raw images were converted into 8-bit grayscale PNG format, varying in size across different patients. The spatial resolutions ranged from 224 × 154 to 512 × 428, and the *Z* direction depth ranged from 6 to 18. All individual raw MRI data were normalized using their mean and SD of color intensity. To optimize the training of our network, we undertook specific preprocessing and data augmentation measures. For the LA dataset, the challenge was the minimal proportion of positive voxels, making it tough to train the network directly with these data. To address this, we resized and center-cropped each 3D volume to exclude the background’s outer edges. Furthermore, we implemented random cropping to create 128 × 128 × 32 patches. This step was crucial for mitigating the imbalance between positive and negative voxels while simultaneously reducing the demand on graphics processing unit (GPU) memory and computational resources. For the ACDC dataset, the preprocessing step was simpler, involving resizing the inputs to a consistent dimension of 256 × 256. Our data augmentation tactics included applying random rotations and flips to both datasets, along with color jittering for the LA dataset. These methods aim to increase the sample variety, aiding in the reduction of overfitting.

Training schedule: All the experiments were performed on a deep learning server featuring Intel Xeon Gold 6230 CPU, 251 GB RAM, and 6 NVIDIA GeForce RTX 3090 GPUs (24 GB). The models were developed based on Python. The model weights were randomly initialized, and the network was trained from scratch using the adaptive moment estimation (Adam) optimizer. The batch size is set to 4 for the LA dataset and 24 for the ACDC dataset, while the number of epochs is set to 240 for LA and the iterations are set to 30,000 for ACDC. The base learning rate is set 1 × 10^−4^ for LA and 5 × 10^−4^ for ACDC with a cosine annealing scheduler with the maximum number of iterations equal to training epochs and minimal learning rate 1 × 10^−7^, and weight decay is set 1 × 10^−4^ for both LA and ACDC. In accordance with [7], the self-supervised loss is weighted by a Gaussian ramp-up weight, expressed as *δ* =  exp (−5(1 −  min (*t*, *T*_ramp_)/*T*_ramp_)^2^), where *t* denotes the current training step and *T*_ramp_ represents the duration of the ramp-up period, equivalent to one quarter of the total training iterations. In each training batch, we endeavored to maintain an even distribution of labeled and unlabeled data. Specifically, for the LA dataset, we duplicated the labeled data to match the volume of unlabeled data, followed by random sampling from the full dataset. In contrast, for the ACDC dataset, we utilized a method called TwoStreamBatchSampler [6], which allows for the sampling of an arbitrary mix of labeled and unlabeled data in each batch, promoting a balanced learning environment.

### Statistical analysis

Following common practice [5,6], Dice score, intersection over union (IoU) score, 95% Hausdorff distance (95HD), and average surface distance (ASD) were used to assess the segmentation results of each model with the manual labels.

Given 2 masks *M1* and *M2*, Dice score [i.e., *Dice*(*M*1, *M*2)], also called Dice similarity coefficient (DSC), is defined as:

$$\text{Dice}\left(M1,M2\right)=\frac{2|M1\cap M2|}{|M1|+|M2|}$$ (8)

IoU score [i.e., *IoU*(*M*1, *M*2)], also called Jaccard score, is defined as:

$$\mathrm{IoU}\left(A,B\right)=\frac{|M1\cap M2|}{|M1\cup M2|}$$ (9)

95HD [i.e., 95*HD*(*M*1, *M*2)] is defined as:

$$95\mathrm{HD}\left(M1,M2\right)=\max\left(h\left(M1,M2\right),h\left(M2,M1\right)\right)$$ (10)

$$h\left(X_{1},X_{2}\right)=\max_{x1\in X_{1}}\left\{\min_{x2\in X_{2}}\|x1-x2\|\right\}$$ (11)

The 95HD resembles the maximum HD but is calculated using the 95th percentile of distances between boundary points in *M1* and *M2*. Therefore, the max function in Eq. 11 represents the highest 5% of these distances.

ASD [i.e., *ASD*(*M*1, *M*2)] is defined as:

$$\begin{array}{c} \mathrm{ASD}\left(M1,M2\right)=\frac{1}{s\left(M1\right)+s\left(M2\right)}\\ \left(\sum_{m1\in M1}‍\min_{m2\in M2}\|m1-m2\|+\sum_{m2\in M2}‍\min_{m1\in M1}\|m2-m1\|\right) \end{array}$$ (12)

where *s*(·) denotes the length of the mask contour.

In summary, the Dice score and IoU score are metrics used to assess the level of overlap between predicted areas and manually labeled areas, emphasizing the internal consistency of predictions. A higher score indicates better prediction accuracy. Conversely, the 95HD and ASD metrics measure the distance between predicted results and manual labels, with a particular focus on segmentation boundaries. In this context, smaller values signify more accurate delineation.

## Results

In contrast to the LA dataset, the ACDC dataset typically comprises fewer MRI slices. As a result, prevailing research commonly employs 2D models for ACDC while opting for 3D models in the case of LA. In this study, we developed our approach utilizing 3D-UNet [26] for LA and UNet [27] for ACDC.

### Comparison with other methods

Comparative performance of our method on LA: The comparative performance of various models on the LA dataset is detailed in Table 1. The efficacy of the supervised 3D-UNet severely relies on densely annotated data, evident in its substantial performance decline from a 91.5% to 80.1% Dice score when labeled data reduce from 100% to 10%. In contrast, our method demonstrates a marked enhancement in the 3D-UNet’s performance with just 10% labeled data. Specifically, 3D-UNet equipped with our approach achieves a 91.0% Dice score and a 84.2% IoU score, showcasing an improvement of +9.9%/16.6% over the standard 3D-UNet. In another setting, where only 20% labeled data are available, our method yields even better results compared to a standard 3D-UNet trained on the complete labeled dataset. It exhibits a noteworthy +8.7%/+13.3% enhancement over the basic 3D-UNet in terms of Dice score and IoU score, respectively. Additionally, it shows a modest increase of +0.6%/+2.1% compared to the full-set 3D-UNet. Notably, our approach emerges as the superior performer across both 20% and 10% labeled data settings compared to other methods, consistently delivering the best results overall.

**Table 1.** Performance comparisons of different methods on the LA dataset. The best results are highlighted in bold font. “-” for SAM-Med2D [28] means that this model has been pre-trained using LA, while “-” for other methods denotes that they did not report the corresponding results. Notably, BCP [44], ACTION [45], and ACTION++ [46] did not report results on the setting of 20% labeled data.

Method	Scans used		Metrics			
	Labeled	Unlabeled	Dice	IoU	95HD	ASD
3D-UNet [26]	80 (100%)	0	0.915	0.832	3.89	1.25
3D-UNet [26]	16 (20%)	0	0.834	0.720	8.97	2.48
3D-UNet [26]	8 (10%)	0	0.801	0.676	11.40	3.27
SAM [29]	80 (100%)	0	0.892	0.808	6.39	2.41
SAM [29]	0	0	0.794	0.660	13.80	4.88
MedSAM [30]	80 (100%)	0	0.868	0.770	7.23	2.54
MedSAM [30]	0	0	0.674	0.512	14.06	5.10
SAM-Med2D [28]	-	-	0.864	0.763	8.25	2.89
UA-MT [8]			0.889	0.802	7.32	2.26
SASSNet [9]			0.895	0.812	8.24	2.20
Double-UA [10]			0.897	0.814	7.04	2.03
Tripled-UA [11]			0.893	0.810	7.42	2.21
CoraNet [12]			0.887	0.811	7.55	2.45
Rec. Lea. [13]	16 (20%)	64 (80%)	0.901	0.820	6.70	2.13
DTC [14]			0.894	0.810	7.32	2.10
3D Grap. [15]			0.898	0.817	6.68	2.12
LG-ER-MT [16]			0.896	0.813	7.16	2.06
GBDL [5]			0.894	0.822	4.03	1.48
SimCVD [47]			0.909	0.838	6.03	1.86
CMM [48]			0.900	-	-	2.40
Ours			**0.921**	**0.853**	**2.91**	**0.98**
UA-MT [8]			0.843	0.735	13.83	3.36
SASSNet [9]			0.873	0.777	9.62	2.55
Double-UA [10]			0.859	0.758	12.67	3.31
Tripled-UA [11]			0.868	0.768	10.42	2.98
CoraNet [12]			0.866	0.781	12.11	2.40
Rec. Lea. [13]	8 (10%)	72 (90%)	0.862	0.760	11.23	2.66
DTC [14]			0.875	0.782	8.23	2.36
3D Grap. [15]			0.879	0.789	8.99	2.32
LG-ER-MT [16]			0.855	0.751	13.29	3.77
GBDL [5]			0.884	0.792	5.89	1.60
SimCVD [47]			0.890	0.803	8.34	2.59
CMM [48]			0.860	-	-	3.24
BCP [44]			0.896	0.813	6.81	1.76
ACTION [45]			0.887	-	-	2.1
ACTION++ [46]			0.899	-	-	1.74
Ours			**0.910**	**0.842**	**3.28**	**1.02**

Comparative performance of our method on ACDC: Table 2 shows the comparison results of different models on the ACDC dataset. Different from the LA dataset, current methodologies usually report results for settings where 10% and 5% labeled data are available. Notably, our method consistently enhances the performance of the basic UNet model across both settings, consistently delivering superior overall results. Again, it is worth noting that our approach achieves top performance in all aspects among other MRI segmentation methods.

**Table 2.** Performance comparisons of different methods on the ACDC dataset. The best results are highlighted in bold font. “-” for MedSAM [30] and SAM-Med2D [28] means that these models have been pre-trained using ACDC, while “-” for other methods denotes that they did not report the corresponding results.

Method	Scans used		Metrics			
	Labeled	Unlabeled	Dice	IoU	95HD	ASD
UNet [27]	70 (100%)	0	0.914	0.846	4.30	0.99
UNet [27]	7 (10%)	0	0.794	0.681	9.35	2.70
UNet [27]	3 (5%)	0	0.478	0.370	31.16	12.62
SAM [29]	70 (100%)	0	0.861	0.768	1.25	0.31
SAM [29]	0	0	0.636	0.491	4.18	1.35
MedSAM [30]	-	-	0.516	0.365	4.95	0.94
SAM-Med2D [28]	-	-	0.869	0.775	1.29	0.36
UA-MT [8]			0.817	0.706	6.88	2.02
SASSNet [9]			0.845	0.743	5.42	1.86
DTC [14]			0.843	0.739	12.81	4.01
URPC [49]	7 (10%)	63 (90%)	0.831	0.724	4.84	1.53
MC-Net [50]			0.864	0.770	5.50	1.84
SS-Net [6]			0.868	0.777	6.07	1.40
CNN&Trans [51]			0.864	-	8.6	-
Ours			**0.893**	**0.812**	**3.77**	**1.04**
UA-MT [8]			0.460	0.360	20.08	7.75
SASSNet [9]			0.578	0.461	20.05	6.06
DTC [14]			0.569	0.457	23.36	7.39
URPC [49]	3 (5%)	67 (95%)	0.559	0.446	13.6	3.74
MC-Net [50]			0.629	0.523	7.62	2.33
SS-Net [6]			0.658	0.554	6.67	2.28
CNN&Trans [51]			0.656	-	16.2	-
Ours			**0.702**	**0.631**	**4.25**	**1.82**

Comparative performance of our method with foundation models: We also conduct comparisons with the recently popular foundation models exemplified by the SAM series [28–30]. Following the approach of MedSAM [30], we fine-tune these foundation models utilizing the bounding box prompts based on labeled data. It is worth noting that SAM-Med2D [28] has been pre-trained on both the LA and ACDC datasets; thus, we refrain from further fine-tuning SAM-Med2D on these datasets. Similarly, we opt not to fine-tune MedSAM using the ACDC dataset, given its prior pre-training on this dataset. Our results, as presented in Tables 1 and 2, consistently demonstrate the superiority of our method over all foundation models, even when utilizing only 10% of labeled data. This holds true even in scenarios where SAM and MedSAM are fine-tuned using the entirety of the labeled data.

### Ablation study

In Table 3, the performance breakdown of our approach in ablation experiments is presented. These experiments were conducted on the LA dataset under settings with 20% and 10% labeled data availability, respectively. Our method comprises 3 integral streams: consistency regularization, pseudo-labeling, and reconstruction. Notably, the absence of any stream results in a performance decline across all evaluation metrics in both settings. It is evident that the complete model consistently achieves the highest performance across all metrics in comparison to individual stream exclusions.

**Table 3.** Ablation studies of different components in our method, without the teacher–student architecture, on the LA dataset. The best results are highlighted in bold font.

Method	Scans used		Metrics			
	Labeled	Unlabeled	Dice	IoU	95HD	ASD
W/o Con.Reg.	16 (20%)	64 (80%)	0.889	0.802	7.32	2.26
W/o Pse.			0.895	0.812	8.24	2.20
W/o Rec.			0.897	0.814	7.04	2.03
Ours			**0.903**	**0.826**	**3.07**	**1.00**
W/o Con.Reg.	8 (10%)	72 (90%)	0.843	0.735	13.83	3.36
W/o Pse			0.873	0.777	9.62	2.55
W/o Rec.			0.859	0.758	12.67	3.31
Ours			**0.894**	**0.811**	**3.34**	**1.02**

Con.Reg., consistency regularization; Pes., pseudo-labeling; Rec., reconstruction

To verify the robustness of our approach, we train our model 10 times with different sampled semi-supervised data settings, and the margin of error is presented by the SD across these diverse runs. The results in Table 4 show the robustness of our approach.

**Table 4.** Margin of errors (SD) on the LA and ACDC datasets

Dataset	Scans used		Metrics			
	Labeled	Unlabeled	Dice	IoU	95HD	ASD
LA	16 (20%)	64 (80%)	0.004	0.006	0.31	0.15
	8 (10%)	72 (90%)	0.004	0.007	0.35	0.11
ACDC	7 (10%)	63 (90%)	0.005	0.008	0.44	0.12
	3 (5%)	67 (95%)	0.012	0.019	0.41	0.13

In Table 5, we examine the impacts of the teacher–student architecture. Evidently, the performance combined with the teacher–student architecture outperforms its non-teacher–student counterpart in Dice by 1.8% and 1.6% on the 20% and 10% labeled data settings, respectively.

**Table 5.** Ablation studies of the teacher–student architecture on the LA dataset. The best results are highlighted in bold font.

Teacher–student	Scans used		Metrics			
	Labeled	Unlabeled	Dice	IoU	95HD	ASD
✓	16 (20%)	64 (80%)	0.903	0.826	3.07	1.00
			**0.921**	**0.853**	**2.91**	**0.98**
✓	8 (10%)	72 (90%)	0.894	0.811	3.34	1.02
			**0.910**	**0.842**	**3.28**	**1.02**

### Qualitative evaluation

**Fig. 5.**
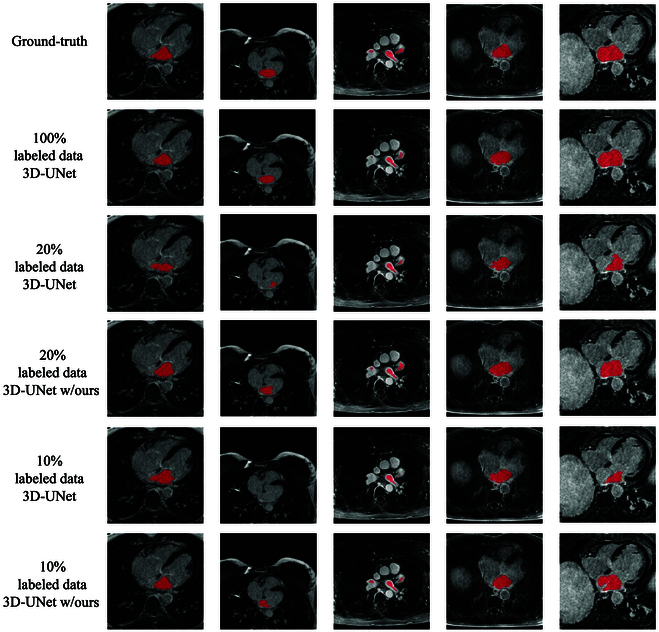
We conducted a segmentation comparison on the LA dataset among multiple scenarios: 3D-UNet with 100% labeled data, 3D-UNet with 20% labeled data, 3D-UNet with 20% labeled data integrated with our method, 3D-UNet with 10% labeled data, and 3D-UNet with 10% labeled data enhanced by our method.

Figure 5 presents a comparative analysis between segmentations generated by 2 kinds of models: the 3D-UNet trained with varying amounts of labeled data and the 3D-UNet integrated with our method specifically designed for scenarios with limited labeled data (20% and 10% availability).

When only 20% of labeled data are accessible, the performance of the 3D-UNet noticeably diminishes in contrast to its performance when trained on a complete set of labeled data (as seen in the third row of Fig. 5). However, our method integrated into the 3D-UNet demonstrates the capability to predict segmentations that closely resemble those produced by the 3D-UNet trained based on 100% of labeled data (as depicted in the fourth row of Fig. 5).

In the more challenging scenario where only 10% labeled data are available, our approach substantially improves the performance of the 3D-UNet (as depicted in the last 2 rows of Fig. 5), showcasing its effectiveness in enhancing segmentation outcomes in scenarios of severely limited labeled data.

### A practical application case: Muscle segmentation

#### Local clinical dataset

Study population: This study was approved by the Medical Ethics Committee of Peking University Third Hospital Review Board (IRB00006761-M2021167). The inclusion criteria: Healthy males aged between 18 and 45 years, with a height ranging from 160 to 175 cm and a BMI (body mass index) within the normal range for males (18.5 to 23.9 kg/m^2^), were enrolled in the study. MRI was performed at baseline (month 0), and follow-up MRI was conducted at the 3rd month and the 9th month after enrollment. The exclusion criteria as follows: (a) MR image was not clear enough to analyze and (b) the volunteers voluntarily withdrew from the experiment.

**Fig. 6.**
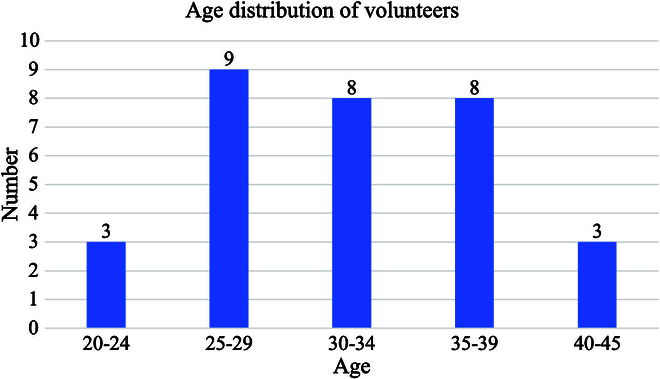
The histogram of age distribution.

Patient characteristics: A total of 37 volunteers’ data, amounting to 111 sets of data, were included in the experiment. Figure 6 illustrates the histogram of age distribution. The average age of the volunteers was 31.65 years, with an average height of 168.51 cm, an average weight of 63.32 kg, and an average BMI of 21.79. The details of patients’ characteristics mentioned above are summarized in Table 6.

**Table 6.** The characteristics of patients

Patient	Cohort (*N* = 37)	
	Mean	SD
Age	31.65	5.21
Hight (cm)	168.51	3.49
Weight (kg)	63.32	6.20
BMI (kg/m^2^)	21.79	2.03

MRI parameters: MRI was performed on a 3.0-T scanner (Discovery 750w, GE Healthcare, WI), using a 3D volumetric interpolated spoiled gradient echo (3D-VIBE) sequence with T1-weighted imaging and Dixon technique for fat-water signal separation to obtain T1-weighted fat-suppressed images. The sequence parameters are as follows: repetition time (TR): 5.4 ms; echo time (TE): 2.1 ms; flip angle: 12^∘^; slice thickness: 4 mm; scanning field of view: 420 mm; reconstruction matrix size: 512 × 512; bandwidth: 558 Hz/voxel. A GEM (gradient echo with multi-shot acquisition) matrix coil was used, with the scanning field of view extending from the anterior superior iliac spine to the calcaneus, and an axial section was acquired in segments. Each acquisition consisted of 100 slices (approximately 40 cm), with an overlap of more than 15% between adjacent acquisition ranges.

Dataset split: There are 255 MR images in total, and muscle groups were delineated in 10 manual regions of interest (ROIs) as detailed in Table 7. For training and validation, we selected 180 and 30 MR images, respectively, with an average of 70.42% of 2D slices labeled in each image. The remaining 45 MR images, each fully labeled, constituted the test set.

**Table 7.** Description of each ROI in the local dataset

S.R.	R. 1	R. 2	R. 3	R. 4	R. 5	R. 6	R. 7	R. 8	R. 9	R. 10
Name	Rectus femoris	Vastus intermedius	Vastus lateralis	Vastus medialis	Semi-endinosus	Soleus	Gastrocnemius	Flexor hallucis longus	Tibialis posterior	Flexor digitorum longus

S.R., symbolic representation; R., ROI

#### Implementation details

Input processing: We implemented the spatial transformation component, which encompasses elastic deformation [27], random cropping, and random rotation. The cropping dimensions were established at 64 × 224 × 224. Subsequently, we applied voxel-wise transformations as described in [7].

Training details: We employ the Adam optimizer, initiating an epoch, which is warmed up by a linear learning rate, followed by a cosine annealing schedule across 15 epochs. The base learning rate is set at 1 × 10^−4^, and the batch size is set at 2. Additionally, the self-supervised loss is weighted by a factor as described in [7]: *δ* =  exp (−5(1 −  min (*t*, *T*_ramp_)/*T*_ramp_)^2^), where *t* represents the current training step and *T*_ramp_ is the duration of the ramp-up period, equivalent to one-quarter of the total training iterations.

#### Experimental results

**Fig. 7.**
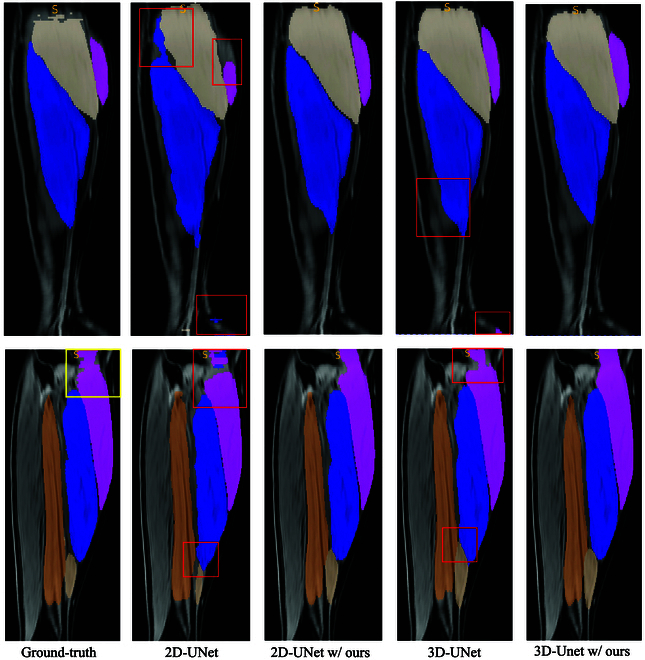
Comparison between 2D-UNet and 3D-UNet, alongside their counterparts enhanced by our approach, on the local clinical dataset. In the visual representation, the yellow box denotes areas lacking manual annotation, while the red box highlights regions where the vanilla models exhibit poorer performance compared to their enhanced counterparts with our method.

We perform experiments using both the 2D and 3D versions of the UNet [26,27] as our baseline. As shown in Table 8, when equipped with our method, 2D-UNet and 3D-UNet achieve 87.3% and 89.5% Dice scores, which are +2.8%/+3.3% higher than their counterparts. Besides, 3D-UNet equipped with our method achieves 92.8% Dice score, delivering the best performance. We also provide a qualitative analysis as shown in Fig. 7. It is evident that both the 2D-UNet and 3D-UNet, enhanced with our method, exhibit superior performance compared to their standard counterparts. Particularly noteworthy is our method’s ability to predict missing annotations, as demonstrated in the second row, even when some manual annotations are absent.

**Table 8.** Performance comparisons of different methods on the local clinical dataset are presented, with results reported as average Dice scores. The best results are highlighted in bold font.

Methods	Dice	R. 1	R. 2	R. 3	R. 4	R. 5	R. 6	R. 7	R. 8	R. 9	R. 10
2D-UNet [27]	0.873	0.933	0.882	0.917	0.844	0.889	0.926	0.878	0.851	0.848	0.758
2D-UNet w/ ours	0.901	0.920	0.909	0.924	0.865	0.907	0.943	0.896	0.887	0.910	0.847
3D-UNet [26]	0.895	0.927	0.870	0.926	0.867	0.905	0.931	0.899	0.885	0.891	0.852
3D-UNet w/ ours	**0.928**	0.974	0.920	0.948	0.921	0.938	0.946	0.917	0.911	0.924	0.885

## Discussion

In this study, a novel semi-supervised MRI segmentation method was proposed and validated across 2 distinct public datasets and one private dataset. Notably, when the labeled data are limited, our approach enables 3D-UNet/UNet to deliver competitive performance in contrast to the counterparts trained on the full label set.

Semi-supervised MRI segmentation models aim to minimize dependence on labeled data while maintaining commendable performance levels. The essence of these models lies in effectively harnessing a significant volume of unlabeled data. In contrast to existing semi-supervised MRI segmentation approaches, our proposed method excels in its intricate utilization of unlabeled data across various dimensions. Primarily, we adopt a pseudo-labeling strategy to derive pseudo-labels for unlabeled MRI scans. These pseudo-labels function akin to ground-truths, empowering the model to glean insights from these supplementary data. Numerous computer vision studies have proven that the integration of unlabeled data through pseudo-labeling typically results in performance enhancements [6,13,31–37], because the model gains a broader perspective from the expansive unlabeled dataset, learning more generalized features that might not be present in the smaller labeled dataset. Second, we introduced a straightforward yet efficient consistency regularization stream to bolster the performance. Consistency regularization aims to make the model’s predictions robust and stable by ensuring that similar inputs (either from augmented versions of the same data or different views of the same instance) produce similar predictions [7,8,10,32,38–42]. It encourages the model to learn more generalizable representations that are less sensitive to minor input alterations. Last, we proposed a reconstruction stream to further explore the distribution of the training data. In semi-supervised learning, generative techniques involve training models to generate new data samples that resemble the distribution of the original dataset. These methods use generative models, such as variational autoencoders (VAEs) [17,43], to accomplish this feat. We build our reconstruction module based on a conditional variational autoencoder (cVAE) [17], enforcing the model generating synthetic samples that simulate the input samples conditioned on those inputs. By capturing the statistical properties of the dataset, our reconstruction stream assists the model in discerning the underlying data distribution.

Benefiting from the design of different strategies for leveraging unlabeled data, UNet/3D-UNet equipped with our approach could achieve competitive performance in contrast to the counterparts that trained in the full labeled set when the quantity of labeled data is scarce (e.g. only 10% labeled MRI scans are available). Besides, our approach achieves the best overall performance among the current semi-supervised MRI segmentation methods. As shown in Results, each of our proposed strategies to harness unlabeled data has been proven to be effective and the synergy between these strategies is evident, with each addition enhancing the performance of the others. This strongly confirms our hypothesis that different strategies for leveraging unlabeled data can complement each other.

### Limitations

First, our model was not tested on MRI scans of a wide range of tissues, as the current popular public benchmarks used in this study are all about the heart and the private dataset is about the muscle. However, our method should have a broad applicability without requiring major changes, because the technologies proposed in this study are independent of segmented objects. Therefore, future research endeavors could prioritize adapting our network for segmenting MRI scans of different tissues.

Second, while our study primarily focused on UNet/3D-UNet, our method has the potential to integrate with various encoder–decoder architectures by adapting certain parameters. Hence, there remains an interest in exploring how our approach could enhance a broader spectrum of MRI segmentation models.

Finally, there are potentially more semi-supervised technologies under exploration, such as semi-supervised generative adversarial network (GAN)-based methods. Initial findings in this study demonstrate the mutual reinforcement among different semi-supervised learning strategies. Therefore, we envision that purposefully designing additional streams of semi-supervised technology can further amplify the performance of our method.

## Conclusion

This study developed a novel semi-supervised MRI segmentation method that explores unlabeled data in various aspects. Our findings demonstrate the effective synergy between distinct semi-supervised technologies, significantly reducing the dependence of UNet/3D-UNet on annotated data while preserving satisfactory performance levels.

## Data Availability

The ACDC and LA datasets are available at https://www.creatis.insa-lyon.fr/Challenge/acdc/databases.html and https://www.cardiacatlas.org/atriaseg2018-challenge/atria-seg-data/, respectively. Our codes are available at https://github.com/yk-pku/Multiple_Aspects.
